# Narrow band imaging for thoracic endometriosis

**DOI:** 10.1186/s40792-020-01000-x

**Published:** 2020-09-30

**Authors:** Takehiro Yamamoto, Ryo Fujikawa, Yoshifumi Arai, Toru Nakamura

**Affiliations:** Departments of General Thoracic Surgery and Pathology, Seirei Hamamatsu General Hospital, 2-12-12 Sumiyoshi, Nakaku, Hamamatsu, Shizuoka 430-8558 Japan

**Keywords:** Thoracic endometriosis, Catamenial pneumothorax, Narrow band imaging (NBI)

## Abstract

**Background:**

The thoracic cavity is the most frequent site of extrapelvic endometriosis. It exhibits a wide variety of clinical manifestations, such as chest pain, cough, and respiratory distress, and is frequently associated with pelvic endometriosis. Although histological confirmation is the gold standard for a definitive diagnosis, endoscopic identification of the affected area is often difficult. Narrow band imaging (NBI) is an imaging technique that emphasizes vascular structures and is reported to be useful in the diagnosis of pelvic endometriosis.

**Case presentations:**

A 31-year-old woman and 39-year-old woman developed a recurrent right pneumothorax during their menstruation cycles. They both had no medical history suggesting pelvic endometriosis. We planned an elective video-assisted thoracoscopic surgery for the suspicion of thoracic endometriosis. In addition to white light alone, an NBI observation enhanced the microvasculature of the suspected lesions and allowed us to identify the affected area more clearly. Partial resections of the diaphragm were performed. Histopathological and immunohistochemical studies of each specimen confirmed the diagnosis of extrapelvic endometriosis.

**Conclusions:**

NBI may improve the diagnostic accuracy for thoracic endometriosis, especially in clinically suspected patients but without a history of pelvic endometriosis.

## Background

Extrapelvic endometriosis is caused by ectopic endometrial tissue outside the abdominopelvic cavity [1]. The thoracic cavity is the most frequent site with a wide variety of clinical manifestations such as chest pain, coughing, and respiratory distress [2, 3]. Although histological confirmation is the gold standard for a definitive diagnosis, endoscopic identification of the affected area is often difficult similar to that of pelvic endometriosis [4]. Narrow band imaging (NBI) is an imaging technique that emphasizes vascular structures and has been reported to be useful for the laparoscopic diagnosis of pelvic endometriosis [5]. Here, we report two cases of thoracic endometriosis exhibiting a catamenial pneumothorax successfully diagnosed by NBI.

## Case presentations

### Case 1

**Fig. 1:**
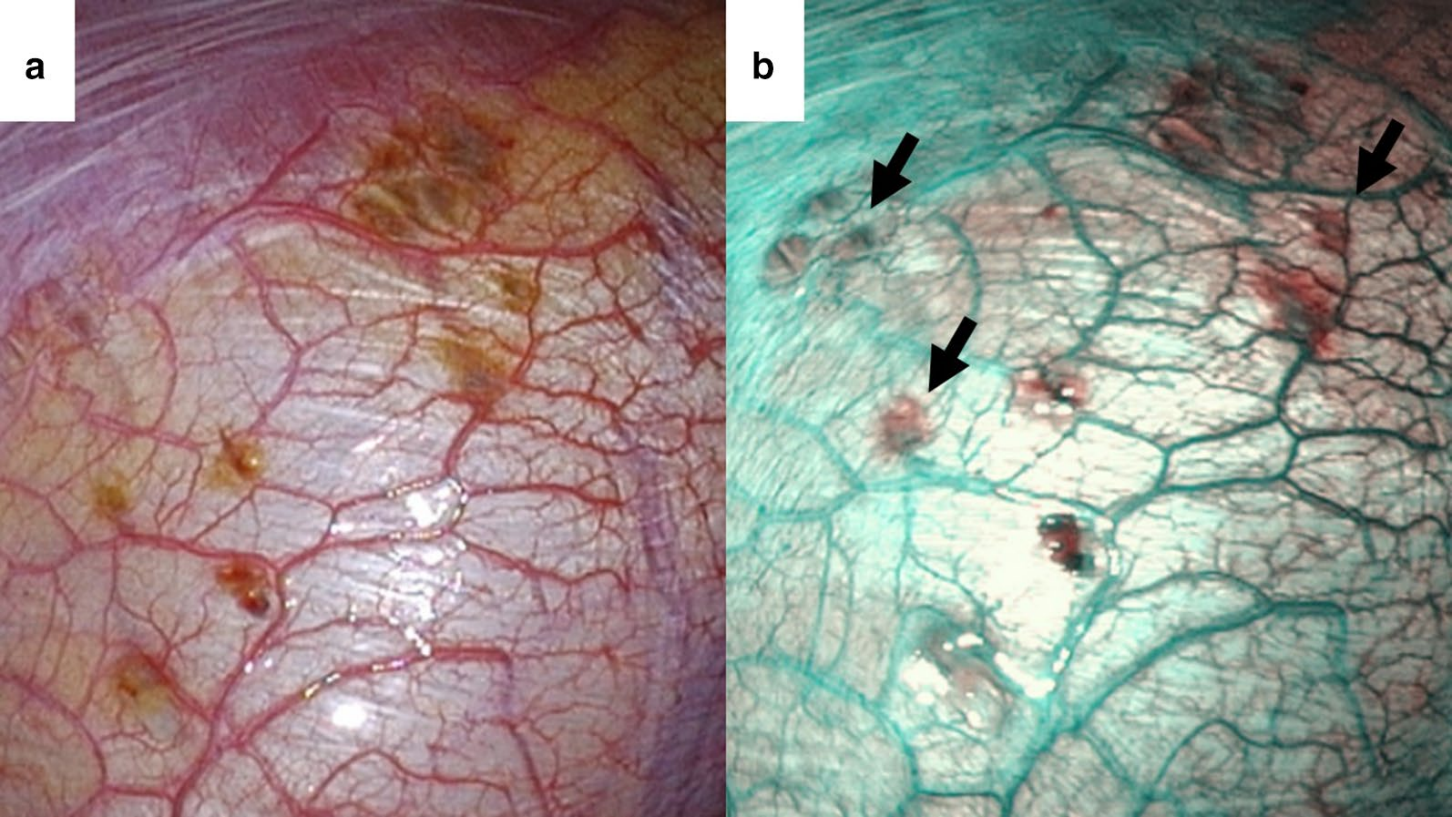
Representative images of the diaphragm in case 1. Some brownish spots were more clearly visualized as dark green with NBI (arrows)

A 31-year-old woman (Gravida 0, Para 0) presented with a recurrent right spontaneous pneumothorax that occurred 4 days after the onset of menstruation. She had a history of an ipsilateral pneumothorax treated by chest tube drainage 2 months prior. Her other past history was negative for dysmenorrhea, pelvic pain, or any other symptoms suggestive of pelvic endometriosis. Given the recurrent pneumothorax without any underlying disease such as a lung cyst on chest computed tomography, we planned an elective video-assisted thoracoscopic surgery (VATS) to rule out thoracic endometriosis during her next menstrual cycle. Under thoracoscopic imaging with an endoscope system (Olympus Endoeye video telescope model LTF-S190-5CE, Olympus Medical Systems Corp., Tokyo, Japan), brownish pleural spots over the centrum tendineum of the diaphragm were observed with standard white light (Fig. 1a). Some of those changes were emphasized and more clearly visualized with dark green in the NBI (Fig. 1b). These morphological changes were judged significant based on a subjective visual inspection. A partial resection of the diaphragm was performed.

### Case 2

**Fig. 2:**
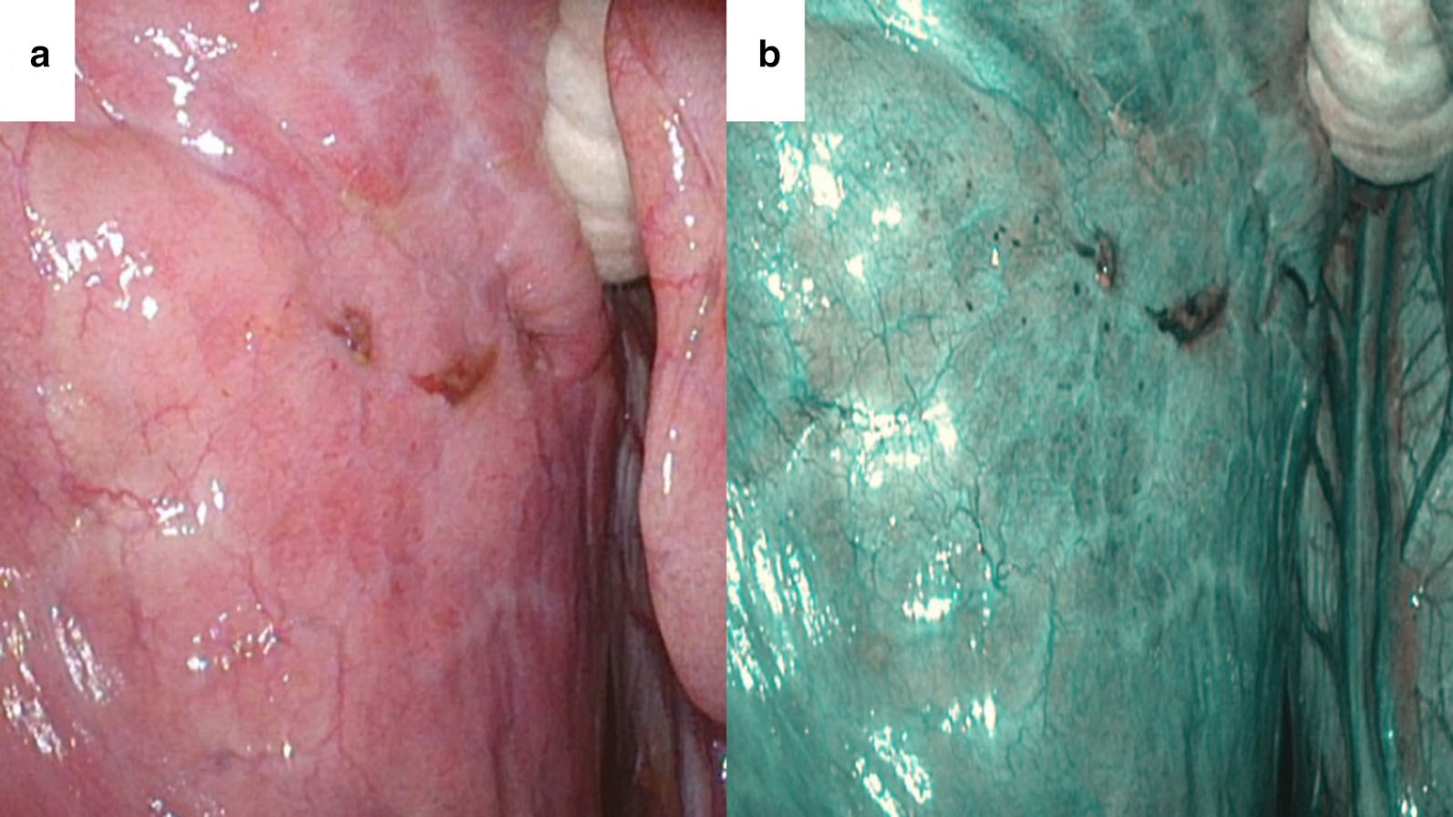
Representative images of the lung surface in case 2. Brownish spots were visualized as dark green with NBI

A 39-year-old woman (Gravida 0, Para 0) presented with a recurrent right spontaneous pneumothorax that occurred a day after the onset of menstruation. She had a history of an ipsilateral pneumothorax that resolved with conservative management 6 months prior. Despite lacking any other history suggesting pelvic endometriosis, we planned an elective VATS for the suspicion of thoracic endometriosis during her next menstrual cycle. Several brownish spots over the lung surface (Fig. 2a) and diaphragm were observed with standard white light. Those changes were visualized as dark green indicating hypervascularity in the NBI (Fig. 2b). A partial resection of the diaphragm was performed.

## Pathological findings and postoperative course

**Fig. 3:**
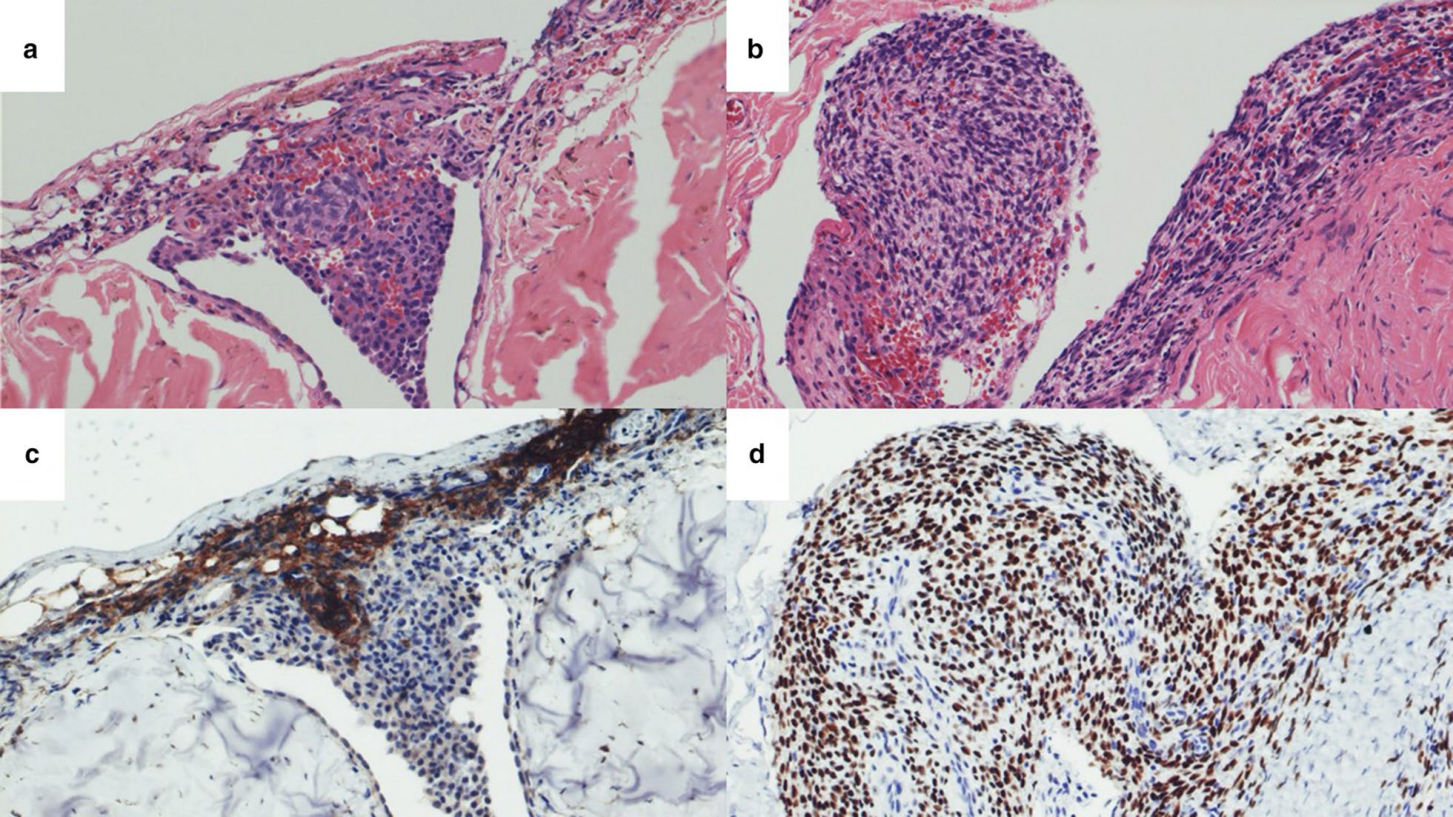
Pathological specimens from both cases showing ectopic endometrial lesions on the surface of the diaphragm, accompanied by inflammatory granulation with hemosiderin-laden macrophages (**a**: Case 1/**b**: Case 2). Immunohistochemical staining of CD10 (**c**: Case 1) and estrogen receptors (**d**: Case 2) with a positive expression that confirms the histological nature of endometriosis

In the histopathological examinations of cases 1 and 2, ectopic endometrial lesions were found on the surface of the pleura and diaphragm and were accompanied by inflammatory granulation with hemosiderin-laden macrophages (Fig. 3a, b). Immunohistochemically, each ectopic endometrial tissue sample was positive for estrogen receptors and CD10 (Fig. 3c, d). Those results were consistent with thoracic endometriosis and a gonadotropin-releasing hormone agonist was administered in both cases. They are currently disease free at 11 months after the surgery.

## Conclusions

Endometriosis is caused by ectopic endometrial tissue in areas other than the uterine cavity, which can lead to a variety of symptoms, such as dysmenorrhea and infertility, and a histological confirmation is the gold standard for a definitive diagnosis [6, 7]. However, the accuracy of the visual identification using laparoscopy is vulnerable depending on the surgeon’s expertise and morphological change in the target lesion during the menstruation cycle [7, 8]. Those factors may lead to a diagnostic delay and poor surgical outcome [9, 10].

The thoracic cavity is the most frequent site of extrapelvic endometriosis [11]. A wide variety of clinical manifestations are seen such as chest pain, coughing, and respiratory distress, and may not necessarily coincide with the menstrual cycle [12]. That may result in diagnostic difficulty even by VATS [1, 13–15]. As with a laparoscopic biopsy for pelvic endometriosis, cyclic changes in the lesion and the skill of the attending surgeon might also influence the outcome [12].

NBI is an imaging technique that emphasizes vascular structures and improves the detection of microvessels not clearly identified under only conventional white light [5]. It is widely used in the gastrointestinal diseases and not costly to perform [16]. Recent studies have reported promising results of NBI for the diagnostic utility of diagnosing pelvic endometriosis by detecting hypervascularity, which is a specific disease pathology [8, 15]. However, to the best of our knowledge, to date, there have been no reports of NBI having been used for thoracic endometriosis.

Although most patients with thoracic endometriosis have been associated with pelvic endometriosis [17], the present cases had no suspicious history before the surgery. Therefore, we applied NBI to improve the diagnostic accuracy at the time of their menstruation cycle. The NBI observation enhanced the microvasculature of the suspected lesions, which was not clearly identified by white light alone, and enabled a histological diagnosis of extrapelvic endometriosis with excellent clinical outcomes.

Our cases demonstrated the effectiveness of NBI for identifying endometrial tissue while obtaining a better surgical view with a more enhanced vascularity than with conventional white light alone. While histological confirmation is still the gold standard of the definitive diagnosis, NBI may improve the diagnostic accuracy of thoracic endometriosis, especially in clinically suspected patients but without a history of pelvic endometriosis.

## Data Availability

Not applicable.

## Abbreviations

VATS: Video-assisted thoracic surgery
NBI: Narrow band imaging
